# Correlation of the Aryl Hydrocarbon Receptor with FSHR in Ovarian Cancer Patients

**DOI:** 10.3390/ijms20122862

**Published:** 2019-06-12

**Authors:** Eileen Deuster, Doris Mayr, Anna Hester, Thomas Kolben, Christine Zeder-Göß, Alexander Burges, Sven Mahner, Udo Jeschke, Fabian Trillsch, Bastian Czogalla

**Affiliations:** 1 Department of Obstetrics and Gynecology, University Hospital, LMU Munich, 81377 Munich, Germany; eileen.deuster@med.uni-muenchen.de (E.D.); anna.hester@med.uni-muenchen.de (A.H.); thomas.kolben@med.uni-muenchen.de (T.K.); christine.zeder-goess@med.uni-muenchen.de (C.Z.-G.); alexander.burges@med.uni-muenchen.de (A.B.); sven.mahner@med.uni-muenchen.de (S.M.); udo.jeschke@med.uni-muenchen.de (U.J.); fabian.trillsch@med.uni-muenchen.de (F.T.); 2 Institute of Pathology, Faculty of Medicine, LMU Munich, 81377 Munich, Germany; doris.mayr@med.uni-muenchen.de

**Keywords:** aryl hydrocarbon receptor (AhR), follicle-stimulating hormone receptor (FSHR), ovarian cancer, immunohistochemistry

## Abstract

Expression of the aryl hydrocarbon receptor (AhR) has been described in various tumor entities from different organs. However, its role in ovarian cancer has not been thoroughly investigated. We aimed to elucidate the prognostic impact of AhR, its correlation with the follicle-stimulating hormone receptor (FSHR), and their functional role in ovarian cancer. By immunohistochemistry, AhR staining was analyzed in a subset of 156 samples of ovarian cancer patients. AhR staining was assessed in the nucleus and the cytoplasm using the semi-quantitative immunoreactive score (IRS), and the scores were grouped into high- and low-level expression. AhR expression was detected in all histological subtypes, with clear cell ovarian cancer displaying the highest staining intensity. Low cytoplasmic expression of AhR was associated with longer overall survival (median 183.46 vs. 85.07 months; *p* = 0.021). We found a positive correlation between AhR and FSHR (*p* = 0.005). Ovarian cancer patients with high cytoplasmic AhR and concurrent FSHR expression had the worst outcome (median 69.72 vs. 43.32 months; *p* = 0.043). Consequently, low cytoplasmic AhR expression seems to be associated with improved survival in ovarian cancer patients. Our data suggest that AhR and FSHR levels correlate with each other, and their concurrent expression was observed in ovarian cancer patients with the worst outcome. Further investigation of the interaction of both receptors and their functional role might better predict the impact of endocrine therapy in ovarian cancer.

## 1. Introduction

Ovarian cancer is one of the five leading types of cancer death in females of all ages with statistics illustrating an increasing rate in Europe [1]. Unfortunately, the five-year survival rate is less than 45% [2]. Ovarian cancer presents itself with non-specific symptoms. This clinical presentation and a lack of screening methods account for ovarian cancer often being diagnosed at a late stage and a consequent worsening of the outcome. 

Some of the most reliable prognostic markers used are the disease stage at diagnosis (FIGO), the volume of residual disease after surgery, high-volume ascites, older age, and histological markers [3,4,5]. However, there are no widely accepted prognostic markers [6,7]. Taking the heterogeneity of ovarian cancer into account is crucial for developing new prognostic and therapeutic strategies. 

The aryl hydrocarbon receptor (AhR) is a ligand-activated transcription factor. It was first identified as the receptor that binds 2,3,7,8-Tetrachlorodibenzo-*p-*dioxin (TCDD), a potent environmental toxicant and carcinogen [8,9]. Upon binding to a ligand in the cytoplasm, the AhR–ligand complex translocates into the nucleus and heterodimerizes with the AhR nuclear translocator (ARNT). The complex binds to the dioxin response elements (DRE), thereby activating a variety of downstream genes and a broad spectrum of biological processes [9]. 

In the ovary, AhR influences ovarian growth and function [10]. It plays an essential role in ovulation by positively affecting the follicle-stimulating hormone receptor’s (FSHR) transcription and thereby altering the follicle-stimulating hormone’s (FSH) responsiveness. Research has shown that AhR-deficient mice (AhRKO) have fewer antral follicles, corpora lutea, and a reduced number of ovulations compared to wild-type (WT) mice [11,12]. In recent years, several studies have analyzed the reasons for this alternated cyclicity focusing on the gonadotropins and their receptors. A close link between AhR and the FSH receptor has been established determining that AhR positively influences mouse FSHR transcription by its direct association through an E-box binding site. Teino et all. showed that the E-box but not USF1 is necessary for AhR binding to the FSHR promoter in vivo. AhR activates the FSHR promoter through the region from −209 to −99 bp [13,14]. 

Furthermore, the effects of AhR in cancer have been extensively discussed with an apparent conflict between its protumorigenic and anti-tumorigenic impacts [9]. In various tumor types, AhR has been shown to be involved in tumor formation and progression [15,16,17,18,19]. Its elevated expression levels in numerous tumor tissues imply its chronical activation [20,21,22]. AhR promotes or inhibits cell growth, and proliferation appears to be dependent on the cell phenotype [8]. 

Due to the scarce amount of knowledge on AhR and its interaction with FSHR in ovarian cancer, an evaluation of AhR expression in different histological subtypes and of its correlation and impact on cancer biology and survival was the primary aim in the current study. 

## 2. Results

### 2.1. Highest Cytoplasmic AhR Staining in Clear Cell Ovarian Cancer 

**Figure 1:**
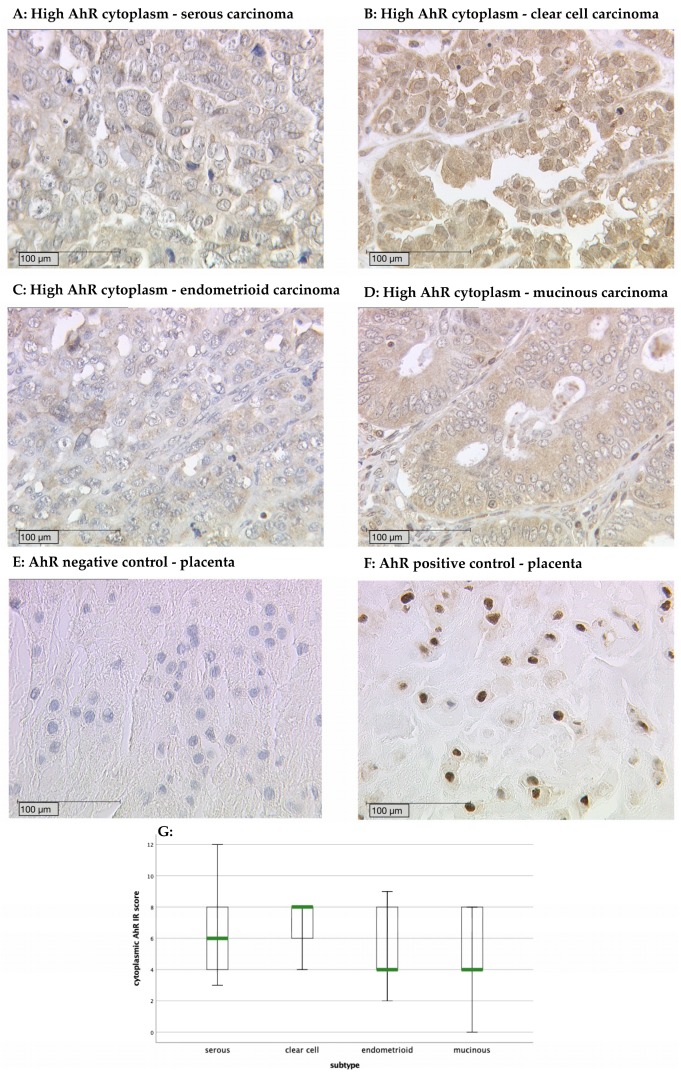
(**A**) High aryl hydrocarbon receptor (AhR) cytoplasm staining (immunoreactive score (IRS) > 3) in ovarian cancer with serous, (**B**) clear cell, (**C**) endometrioid, and (**D**) mucinous histology. (**E**) AhR negative control and (**F**) positive control in human placenta tissue. (**G**) AhR expression in the histological subtypes. The cytoplasmic AhR IR scores were compared between the different histological subtypes. The clear cell cancer specimens tended to have higher IR scores (median = 8) than the other carcinoma types (*p* = 0.077).

Out of 148 successfully stained ovarian cancer specimens, 145 (98%) showed positive nuclear AhR expression. In the cytoplasm, 147 (99%) of cases were positive. The median immunoreactive score (IRS) of the nucleus and cytoplasm was 6 (0–12). When comparing cytoplasmic AhR expression between the different histological subtypes (Figure 1A–D), it becomes apparent that the clear cell carcinoma shows higher IR scores (median IRS = 8 with a range of 4–8; *p* = 0.077) (Figure 1G). 

### 2.2. AhR Expression Correlates with Clinical and Pathological Data

We analyzed the correlation between AhR and clinicopathological data such as histology, grading, nearby lymph nodes (pN), size of primary tumor (pT), and FIGO classification (Table 1). In the cytoplasm, we found a significant negative correlation between high AhR and histology (*p* = 0.000; Rho = −0.296). A positive correlation was observed between high AhR and pT (*p* = 0.028; Rho = 0.159) as well as FIGO (*p* = 0.012; Rho = 0.189). High AhR correlated with high serous grading (*p* = 0.004; Rho = 0.216) and the grading from the other histological subtypes (*p* = 0.002; Rho = −0.236). In the nucleus, AhR significantly correlated with histology (*p* = 0.002; Rho = 0.274), pT (*p* = 0.034; Rho = −0.186), and FIGO (*p* = 0.014; Rho = 0.219). Nuclear AhR and cytoplasmic AhR expression were observed to not correlate with each other (*p* = 0.173; Rho = 0.083). 

### 2.3. AhR Staining Intensity Correlates with FSHR Expression

FSHR expression was observed in 44 of 151 (29.1%) specimens with a median IRS of 3 and a range of 0–12. There were no significant differences in FSHR expression when comparing all histological subtypes (*p* = 0.397). Also, all other parameters such as pT (*p* = 0.099), pN (*p* = 0.451), FIGO (*p* = 0.075), and grading (*p* = 0.314) showed no significant correlation to FSHR expression. 

**Figure 2:**
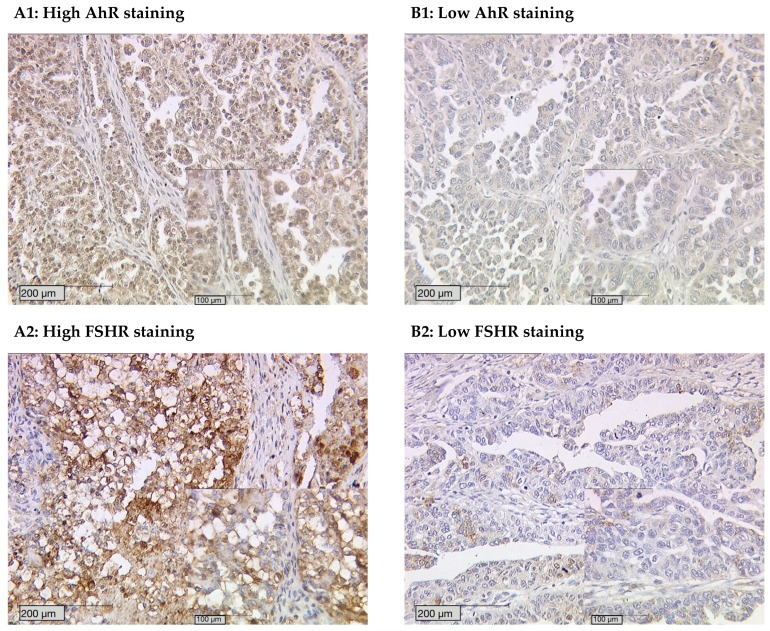
AhR staining intensity correlates with FSH receptor expression. High AhR staining (**A1**) corresponds with high follicle-stimulating hormone receptor (FSHR) staining (**A2**). Low AhR staining (**B1**) correlates to low FSHR (**B2**) found in specimens from the same patients.

However, the examination of both AhR and FSHR revealed that AhR staining intensity is strongly correlated with FSHR expression in the ovarian cancer specimens. Patients with high AhR staining tend to have high FSHR expression levels (Figure 2). 

**Figure 3:**
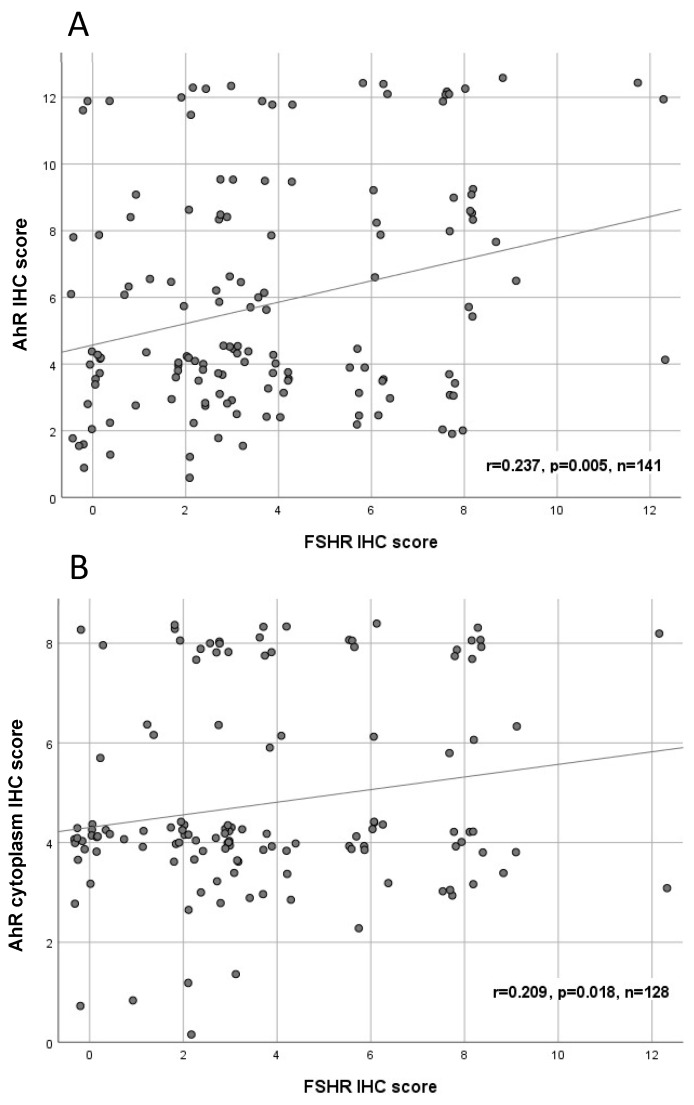
Correlation analysis of AhR (**A**)/cytoplasmic AhR (**B**) and FSHR in ovarian cancer tissue (*n* = 141/128). A significant correlation of cytoplasmic AhR/AhR cytoplasm expression with FSHR expression was noted. For better visualization, dots have been jittered.

By analyzing the different expression levels, we identified significant correlations between AhR expression in general, specific cytoplasmic AhR expression, and FSHR (FSHR and AhR *p* = 0.005, Rho = 0.237; FSHR and high AhR cytoplasm *p* = 0.018, Rho = 0.209) (Table 2, Figure 3). 

### 2.4. Cytoplasmic AhR Expression Impairs Survival on its Own and Affects the Role of FSHR in Survival

The median age of the patients was 62 ± 12 years with a range of 31–88 years. The patients’ median survival time was 51.2 ± 57.6 months and their median months free of recurrence was 56.4 ± 57.6.

**Figure 4:**
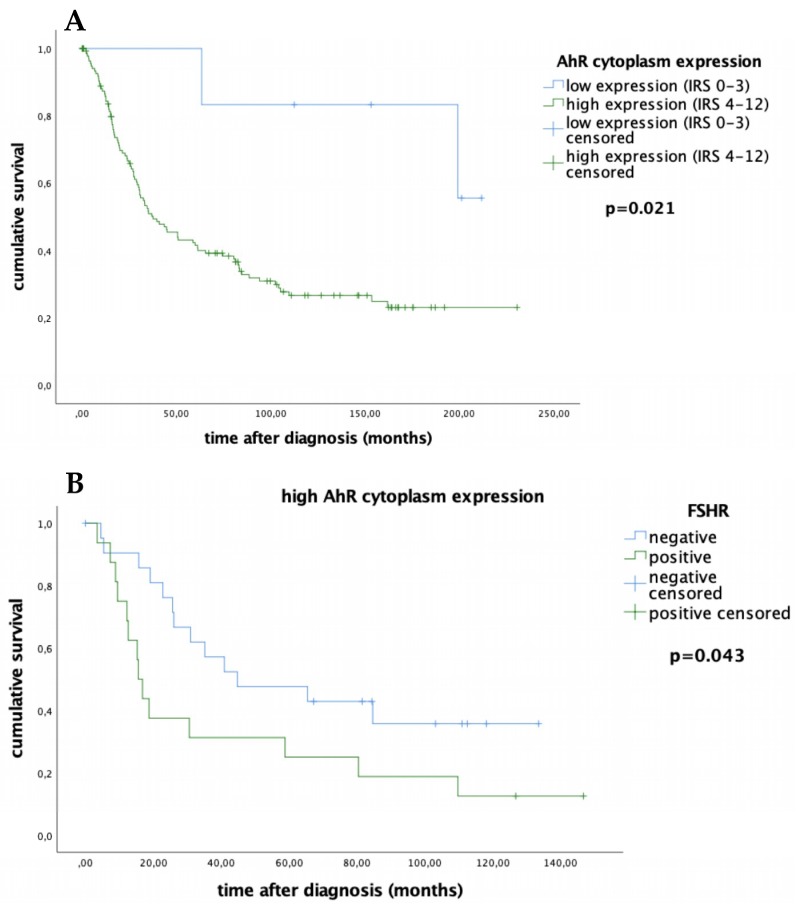
Correlation between AhR expression and overall survival. Ovarian cancer patients with low cytoplasmic AhR expression (IRS 0–3) tend to have better overall survival compared to those with high expression (IRS 4–12) (*p* = 0.021) (**A**). For patients with higher cytoplasmic AhR expression, the amount of FSHR present changes their outcome (**B**).

Low cytoplasmic AhR expression (IRS 0–3) is associated with prolonged overall survival. As depicted on the Kaplan–Meier curve, lower cytoplasmic AhR expression correlates with a significantly better prognosis (Figure 4A, median 183.46 vs. 85.07 months; *p* = 0.021). Nuclear AhR expression has no impact on survival, as shown in supplementary Figure S2 (median 90.74 vs. 89.09 months; *p* = 0.574). 

Cytoplasmic AhR seems to affect the role of FSHR in survival. Patients with high AhR expression (IRS > 4) in the cytoplasm have a worse overall survival if their FSHR levels are positive. However, patients with high cytoplasmic AhR expression and concurrent negative FSHR levels exhibit better overall survival (Figure 4B, median 69.72 vs. 43.32 months; *p* = 0.043). FSHR expression alone has no impact on survival, as shown in supplementary Figure S1 (median 91.08 vs. 80.23 months, *p* = 0.448).

### 2.5. Clinical and Pathological Parameters are Independent Prognostic Factors

Multivariate Cox regression analysis was performed to detect which parameters were independent prognostic factors for overall survival in the present cohort. In this analysis, the patient’s age (*p* = 0.020) and FIGO status (*p* = 0.004) were independent prognostic factors for overall survival. High cytoplasmic AhR (*p* = 0.771) and FSHR (*p* = 0.265) did not prove to be independent prognostic factors. The combination of a high cytoplasmic AhR staining and positive FSHR staining did not show to be of independent impact either (*p* = 0.058). (Table 3). 

## 3. Discussion

As a ligand-activated transcription factor of the carcinogen TCDD, the aryl hydrocarbon receptor (AhR) was identified as a potential oncogenic factor influencing ovarian cancer biology and outcome. By an E-box binding site, AhR mediates transcription of the FSH receptor and might thereby represent a link to the hormonal system in ovarian cancerogenesis. So far, only little is known about the specific role of AhR in ovarian cancer, and this study represents the first investigation focusing on AhR expression in different histological subtypes and its impact on survival.

In recent years, the aryl hydrocarbon receptor’s role in carcinogenesis has often been discussed, elucidating its pro-oncogenic and anti-tumorigenic roles depending on the tumor type [8]. The overexpression of AhR has been described in various cancers including pancreatic, prostate, lung, urinary tract, and esophageal tumors [20,21,23,24,25,26]. Similar to our data, studies have shown increased levels of AhR in different carcinomas to correlate with poor prognosis. In lung squamous cell carcinoma, Su et al. found increased levels of nuclear AhR to be associated with poor prognosis [27]. Similarly, in the upper urinary tract, a correlation between AhR expression and tumor grade was demonstrated [25]. Increased AhR expression and activity is believed to build a pro-inflammatory tumor environment, leading to tumor progression [9]. 

On the other hand, other studies have discussed an anti-tumorigenic effect of AhR. In breast cancer, high levels of AhR expression were inversely correlated with grading [28]. This is suggested to be due to an AhR–estrogen receptor crosstalk. It is well established that AhR antagonizes estrogen receptor activity and vice versa [9]. In breast cancer, there has been extensive research on the role AhR plays in estrogen-dependent breast cancer. In ovarian cancer, however, little is known. Li et al. achieved results implying that TCDD could suppress the proliferation of ovarian cancer cell line OVCAR-3. However, this effect could not be reproduced in other ovarian cancer cell lines [29]. Thus, further studies are needed to understand the mechanisms and effects of AhR pathways by focusing specifically on ovarian cancer and its different histological subtypes.

In our study, we examined the expression of the aryl hydrocarbon receptor in different histological types of ovarian cancer (serous, clear cell, endometrioid, and mucinous) and its association with clinicopathological data and overall survival. We evaluated AhR expression, differentiating between nuclear and cytoplasmic localization, to determine whether the transcriptional function is active or not. The expression of the receptors’ target gene cytochrome P450 1 A1 relies mainly on the activity of AhR through several DREs. 

In this study, low cytoplasmic AhR expression was associated with better overall survival. We were able to show that the immunohistochemical evaluation of cytoplasmic AhR correlated with histology, grading (except in low-grade serous cancer), tumor size, and FIGO. 

These results are consistent with the findings of another study describing a difference in AhR staining in serous epithelial ovarian cancer of low grade when compared to high grade [30]. The elevated expression of AhR in poorly differentiated ovarian cancer is concordant with its activity of inducing the transcription of several enzymes which are crucial for the activation of carcinogens (e.g., polycyclic aromatic hydrocarbons) [9]. 

Furthermore, our study revealed a strong correlation between (cytoplasmic) AhR and FSHR in ovarian cancer patients. This interaction of AhR and FSHR has already been characterized in healthy ovarian follicles. AhRKO follicles were shown to have reduced mRNA levels of gonadotropin receptors compared to WT follicles, thus decreasing gonadotropin responsiveness [13,31]. The aryl hydrocarbon receptor positively influences FSHR transcription by its direct association through an E-box binding site [13,14]. In our study, we found that ovarian cancer patients with high cytoplasmic AhR expression have different survival outcomes depending on their FSHR levels (Figure 4). The patients with high cytoplasmic AhR and FSHR expression had the worst outcome. As previously described, FSHR on its own does not correlate to clinical and pathological data and does not affect the survival outcome in ovarian cancer patients [32]. However, if AhR is expressed in the cytoplasm, FSHR affects survival. 

**Figure 5:**
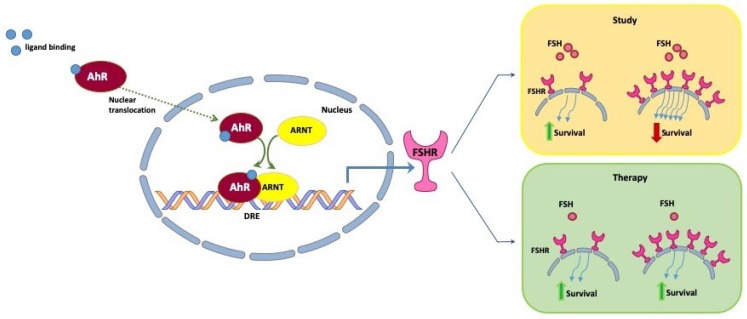
Schematic illustration of the molecular pathway of AhR and its effects on FSHR and survival. Upon binding to a ligand in the cytoplasm, the AhR–ligand compound translocates into the nucleus and heterodimerizes with the AhR nuclear translocator (ARNT). The complex binds to the dioxin response elements (DRE). AhR activates FSHR transcription by binding to the promoter of this gene (not shown in the figure). In our study, depicted in the yellow box, we found that ovarian cancer patients with low FSHR levels had a better outcome than patients with a high number of FSH receptors. In the green box, we wanted to illustrate our hypothesis further. Ovarian cancer patients with high levels of FSHR could benefit from a gonadotropin-releasing hormone (GnRH) modulation therapy as it would lead to reduced FSH levels and thereby potentially reduce the responsiveness of FSHR.

Ovarian cancer patients with high levels of AhR and FSHR could potentially benefit from treatment with gonadotropin-releasing hormone (GnRH) agonists/antagonists. To improve the outcome of these patients, we propose to lower FSH levels in the blood through GnRH agonists/antagonists. The overexpression of FSHR could then potentially not lead to its negative effect due to the lack of ligands. In our study, the highest AhR levels were seen in clear cell ovarian cancer patients. As AhR upregulates FSHR, these patients would possibly benefit the most from this therapeutic approach (Figure 5). Further understanding of the AhR pathway, especially in clear cell ovarian cancer, might open up new therapeutic approaches for this rare histologic subtype.

## 4. Materials and Methods

### 4.1. Patients and Specimens

In this study, ovarian cancer samples from 156 patients who underwent surgery in the period 1990–2002 at the Department of Obstetrics and Gynecology, Ludwig Maximilian University in Munich, Germany were analyzed. The women undergoing surgery for ovarian cancer were between 31 and 88 years old, and their median age was 62 ± 12 years. We only included patients with a definite diagnosis of ovarian cancer in this study; borderline tumors or benign tumors were excluded. None of the patients had neoadjuvant chemotherapy. Clinical data were retrieved from the patients’ charts, and the Munich Cancer Registry (MCR) provided the follow-up data. A pathologist at the Department of Pathology, Ludwig Maximilian University performed the histological classification (serous (*n* = 110), endometrioid (*n* = 21), mucinous (*n* = 13), clear cell (*n* = 12)) and tumor grading. The serous ovarian cancer samples were divided into low and high grading. Endometrioid ovarian cancer was graded according to G1 to G3. For mucinous carcinoma, there is no WHO classification; however, the subtype is often classified into G1 to G3. The clear cell cancer was always categorized as G3. The staging of the tumor was performed using FIGO classification (I = 35, II = 10, III = 103, IV = 3). The TNM classification was derived by assessing the size or direct extent of the primary tumor (T1 (*n* = 40), T2 (*n* = 18), T3 (*n* = 93), and T4 (*n* = 4)); the degree to which it spread to regional lymph nodes (N0 (*n* = 43), N1 (*n* = 52)), which was only known in 95 cases; and by evaluating the 9 cases in which distant metastasis was present (M0 (*n* = 3), M1 (*n* = 6)) (Table 4). 

### 4.2. Ethics Approval

The ovarian cancer specimens were initially collected for histopathological diagnostics. However, at the time of our study, all diagnostic procedures had been fully completed, and the samples were no longer used for clinical tests. The patients’ data were fully anonymized. The current study was approved by the Ethics Committee of the Ludwig-Maximilians-University, Munich, Germany (approval number 227-09) on the 30 September 2009. The Declaration of Helsinki 1975 was respected.

### 4.3. Immunohistochemistry

The formalin-fixed and paraffin-embedded ovarian cancer tissue samples were deparaffinized in Xylol for 20 min. The tumor slides were washed in 100% ethanol. The endogenous peroxidase was blocked with 3% H_2_O_2_/methanol for 20 min. The slides were rehydrated in a descending series of alcohol. Then, the samples were placed in a pressure cooker containing a buffer solution (0.1 M sodium citrate and 0.1 M citric acid pH = 6.0) and cooked for 5 min. The slides were washed in distilled water and PBS buffer. In order to prevent non-specific binding of the primary antibody, a blocking solution was used (ZytoChem Plus HRP Polymer System, Berlin, Germany). The slides were incubated with the AhR primary antibody (anti-AhR antibody polyclonal rabbit IgG; Abnova, PAB18068) at a 1:200 dilution or FSHR primary antibody (anti-FSHR antibody polyclonal rabbit IgG; Novus Biologicals, NLS2231) at a 1:100 dilution for 16 h at 4 °C. After incubation with a corresponding biotinylated secondary anti-rabbit IgG antibody and with the associated avidin–biotin–peroxidase complex (ZytoChem Plus HRP Polymer System, Berlin, Germany), visualization was performed with a substrate (Imidazole-HCl buffer, containing hydrogen peroxide and an anti-microbial agent) and chromogen 3, 3-diamino-benzidine (Dako, Munich, Germany). Finally, the slides were counterstained with hemalum staining (2 min), dehydrated in an ascending series of alcohol, and covered. Negative and positive controls were used to assess the specificity of the immunoreactions (Figure 1E,F). Negative controls (colored in blue) were performed in placental tissue by replacement of the primary antibodies by species-specific (rabbit) isotype control antibodies (Dako, Glostrup, Denmark). For the positive control, placental, vaginal, and intestinal tissues were used.

The intensity of the AhR expression was assessed using the immunoreactive score (IRS). This is a well-established semi-quantitative scoring system. The IRS is obtained by multiplying the staining intensity (negative = 0, weak = 1, moderate = 2, strong = 3) with the percentage of positive cells (negative = 0, ≤10% = 1, ≥11%, ≤50% = 2, ≥51%, ≤80% = 3, ≥81% = 4) resulting in an IR score between 0 and 12. In our study, two independent scorers analyzed the intensity and distribution pattern using a Leitz (Wetzlar, Germany) microscope. For AhR, the cytoplasm and the nucleus were scored independently (AhR cytoplasm and AhR nucleus). In the cytoplasm, a score between 0 and 3 was marked as low, and 4–12 was high. In the nucleus, a score between 0 and 5 was regarded as low, and IRS ≥ 6 was regarded as a high score. The median FSHR receptor expression level (IRS = 3) was used as a cut-off to define FSHR-positive (IRS >3) vs. -negative (IRS ≤ 3) tumors.

### 4.4. Statistical Analysis

All data were collected, processed, and analyzed using IBM SPSS Statistics, version 25.0 (IBM Corporation, Armonk, NY, USA). Values of *p* smaller than 0.05 were considered statistically significant. The distribution of clinical pathological variables was evaluated with the chi-square test. Kaplan–Meier curves and log-rank testing (Mantel–Cox) were performed to compare survival times between different groups. Cut-off points were obtained through the receiver operator curve (ROC). This is a graph in which sensitivity is plotted on the *y* axis and (1 – specificity) is plotted on the *x* axis [33]. The Youden index is defined as (sensitivity + specificity – 1). Its maximum value can be used to select the optimal cut-off point [34,35]. 

Spearman’s correlation test was used to calculate the correlation of immunohistochemical staining results with histopathological data. Cox regression was performed to determine which parameters were independent.

## 5. Conclusions

In this study, we observed that AhR is a prognostic marker of survival. Patients with lower AhR expression in the cytoplasm have a better prognosis. Immunochemical evaluation of AhR correlated with the grading status (except for in low-grade serous cancer). Furthermore, we found that AhR and FSHR levels correlated with each other, and their common expression was associated with an unfavorable outcome.

## Figures and Tables

**Table 1:** Correlations between high cytoplasmic/nuclear AhR expression and clinical data.

	Cytoplasmic AhR Expression		Nuclear AhR Expression	
Variables	*p*	Correlation Coefficient	*p*	Correlation Coefficient
Histology	0.000	−0.296	0.002	0.274
pT	0.028	0.159	0.034	−0.186
pN	0.101	0.136	0.147	−0.162
FIGO	0.012	0.189	0.014	0.219
Grading				
*serous—low grading*	0.475	−0.005	0.551	0.053
*serous—high grading*	0.004	0.216	0.052	−0.170
*clear cell, endometrioid, and mucinous—G1 to G3*	0.002	−0.236	0.679	0.037

**Table 2:** Correlation analysis for AhR, high AhR in the cytoplasm, and FSHR.

Staining	AhR	High AhR Cytoplasm	FSHR
AhR			
cc	1.000	0.395	0.237
*p*	.	0.000	0.005
*n*	145	130	141
high AhR cytoplasm			
cc	0.395	1.000	0.209
*p*	0.000	.	0.018
*n*	130	132	128
FSHR			
cc	0.237	0.209	1.000
*p*	0.005	0.018	.
*n*	141	128	151

IR scores of AhR, AhR in the cytoplasm, and FSHR staining were correlated to each other using Spearman’s correlation analysis. cc = correlation coefficient, *p* = two-tailed significance, *n* = number of patients.

**Table 3:** Multivariate analysis.

Covariate	Coefficient (b_i_)	[HR Exp(b_i_)]	95% CI		*p*-value
			Lower	Upper	
Histology serous clear cell endometrioid mucinous	−0.836 −0.441 −0.287	0.433 0.643 0.750	0.054 0.075 0.178	3.459 5.485 3.161	0.430 0.687 0.696
FIGO (I, II vs. III, IV)	1.097	2.994	1.420	6.315	**0.004**
Grading serous low serous high clear cell, endometrioid and mucinous—G1 to G3	−0.478 1.145 −0.002	0.630 3.141 0.998	0.146 0.889 0.422	2.631 11.101 2.363	0.517 0.076 0.997
Patients’ age (≤60 vs. >60 years)	0.580	1.786	1.095	2.914	0.020
High AhR cytoplasmic	0.074	1.077	0.653	1.776	0.771
FSHR positive	−0.987	0.373	0.066	2.116	0.265
AhR cytoplasmic and FSHR positive	1.651	5.210	0.947	28.646	0.058

**Table 4:** Patient characteristics.

Parameters	N	Percentage
**Histology**		
serous	110	70.5%
clear cell	12	7.7%
endometrioid	21	13.5%
mucinous	13	8.3%
Lymph nodes		
pNX pN0	61 43	39.1% 27.6%
pN1	52	33.3%
Distant Metastasis		
pM0/X	150	96.2%
pM1	6	3.8%
Grading serous		
low	24	23.0%
high endometrioid G1 G2 G3 mucinous G1 G2 G3 clear cell G3	80 6 5 8 6 6 0 9	77.0% 31.6% 26.3% 42.1% 50.0% 50.0% 0% 100%
FIGO		
I II	35 10	23.1% 6.6%
III IV	103 3	68.2% 2.0%
Age		
≤60 years	83	53.2%
>60 years	73	46.8%

## Supplementary Materials

Supplementary materials can be found at https://www.mdpi.com/1422-0067/20/12/2862/s1.

## Abbreviations

AhR	Aryl hydrocarbon receptor
FSH	Follicle-stimulating hormone
FSHR	Follicle-stimulating hormone receptor
IRS	Immunoreactive score
TCDD	2,3,7,8-Tetrachlorodibenzo-*p-*dioxin
