# Supplementary material for: Correlation of the Aryl Hydrocarbon Receptor with FSHR in Ovarian Cancer Patients
Source: Int J Mol Sci. 2019 Jun 12;20(12):2862. doi: 10.3390/ijms20122862 (PMC6628023; doi:10.3390/ijms20122862)
Supplement: Supplementary file 1 [file ijms-20-02862-s001.pdf]

Supplementary Materials:

A

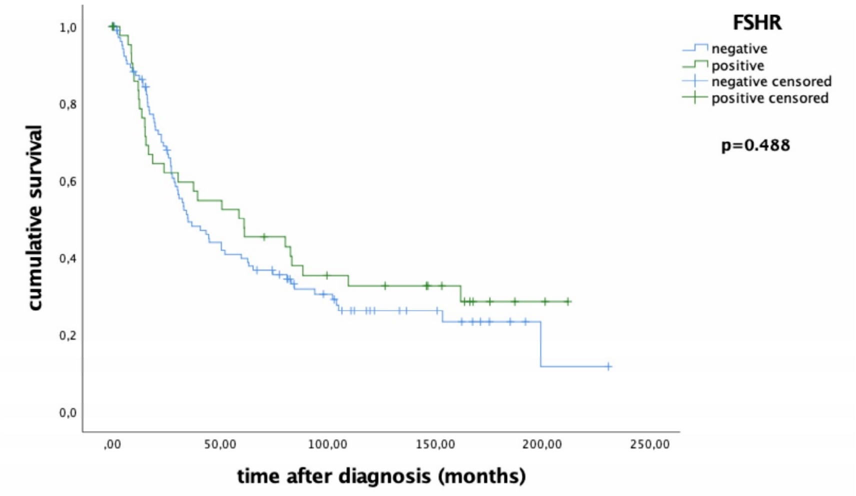

Figure 1: Kaplan-Meier curve: survival of patients with negative vs. positive FSHR expression. There is no significant difference in survival between the two groups (p=0.488).

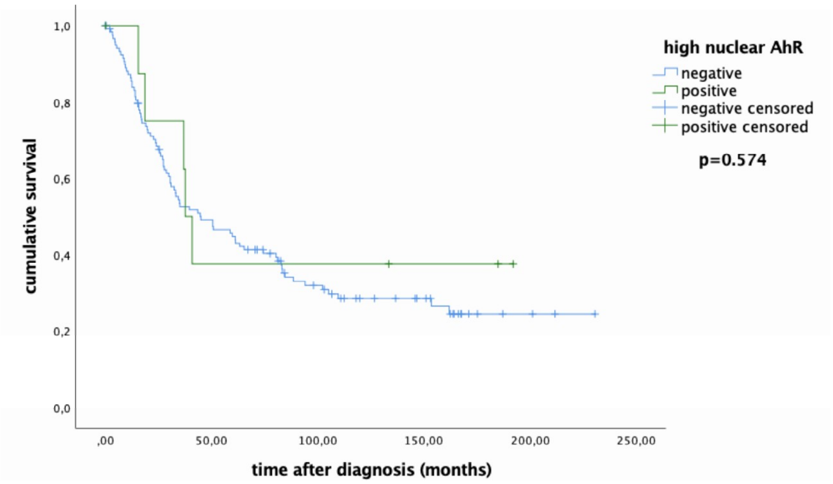

Figure 2: Kaplan-Meier curve: survival of patients with high nuclear AhR staining. There is no significant difference in survival between high and low nuclear intensity (p=0.574).
